# Chemoresistance is associated with increased cytoprotective autophagy and diminished apoptosis in bladder cancer cells treated with the BH3 mimetic (−)-Gossypol (AT-101)

**DOI:** 10.1186/s12885-015-1239-4

**Published:** 2015-04-07

**Authors:** Jens Mani, Stefan Vallo, Stefanie Rakel, Patrick Antonietti, Florian Gessler, Roman Blaheta, Georg Bartsch, Martin Michaelis, Jindrich Cinatl, Axel Haferkamp, Donat Kögel

**Affiliations:** 1 Department of Urology, Goethe University Hospital, Theodor-Stern-Kai 7, D-60590 Frankfurt am Main, Germany; 2 Experimental Neurosurgery, Neuroscience Center, Goethe University Hospital, Theodor-Stern-Kai 7, D-60590 Frankfurt am Main, Germany; 3 Institute for Medical Virology, Goethe University Hospital, Theodor-Stern-Kai 7, D-60590 Frankfurt am Main, Germany; 4 School of Biosciences, The University of Kent, Canterbury, Kent CT2 7NZ UK

## Abstract

**Background:**

Acquired resistance to standard chemotherapy causes treatment failure in patients with metastatic bladder cancer. Overexpression of pro-survival Bcl-2 family proteins has been associated with a poor chemotherapeutic response, suggesting that Bcl-2-targeted therapy may be a feasible strategy in patients with these tumors. The small-molecule pan-Bcl-2 inhibitor (−)-gossypol (AT-101) is known to induce apoptotic cell death, but can also induce autophagy through release of the pro-autophagic BH3 only protein Beclin-1 from Bcl-2. The potential therapeutic effects of (−)-gossypol in chemoresistant bladder cancer and the role of autophagy in this context are hitherto unknown.

**Methods:**

Cisplatin (5637^r^CDDP^1000^, RT4^r^CDDP^1000^) and gemcitabine (5637^r^GEMCI^20^, RT4^r^GEMCI^20^) chemoresistant sub-lines of the chemo-sensitive bladder cancer cell lines 5637 and RT4 were established for the investigation of acquired resistance mechanisms. Cell lines carrying a stable lentiviral knockdown of the core autophagy regulator ATG5 were created from chemosensitive 5637 and chemoresistant 5637^r^GEMCI^20^ and 5637^r^CDDP^1000^ cell lines. Cell death and autophagy were quantified by FACS analysis of propidium iodide, Annexin and Lysotracker staining, as well as LC3 translocation.

**Results:**

Here we demonstrate that (−)-gossypol induces an apoptotic type of cell death in 5637 and RT4 cells which is partially inhibited by the pan-caspase inhibitor z-VAD. Cisplatin- and gemcitabine-resistant bladder cancer cells exhibit enhanced basal and drug-induced autophagosome formation and lysosomal activity which is accompanied by an attenuated apoptotic cell death after treatment with both (−)-gossypol and ABT-737, a Bcl-2 inhibitor which spares Mcl-1, in comparison to parental cells. Knockdown of ATG5 and inhibition of autophagy by 3-MA had no discernible effect on apoptotic cell death induced by (−)-gossypol and ABT-737 in parental 5637 cells, but evoked a significant increase in early apoptosis and overall cell death in BH3 mimetic-treated 5637^r^GEMCI^20^ and 5637^r^CDDP^1000^ cells.

**Conclusions:**

Our findings show for the first time that (−)-gossypol concomitantly triggers apoptosis and a cytoprotective type of autophagy in bladder cancer and support the notion that enhanced autophagy may underlie the chemoresistant phenotype of these tumors. Simultaneous targeting of Bcl-2 proteins and the autophagy pathway may be an efficient new strategy to overcome their “autophagy addiction” and acquired resistance to current therapy.

## Background

Bladder cancer is the second most common genitourinary tumor, and the fourth most common entity of malignancy-related deaths of men in the Western world [1]. The deregulation of apoptosis in various malignancies, including those of the genitourinary tract, supports the entry of more tumor cells into the proliferative cycle [2]. The effects of most of the chemotherapies and radiotherapies are exerted through activation of pro-apoptotic pathways. An interference of those pathways has a severe impact on the formation of drug-resistant, aggressive tumors, which show a worse clinical prognosis [3]. With the genesis of drug resistance in genitourinary cancers, apoptosis has become a prime therapeutic target in the last decade. Recent studies have also shown that the cellular suicide can be executed by non-apoptotic forms of programmed cell death such as necroptosis and autophagic cell death [4,5]. The anti-apoptotic proteins of the Bcl-2 family are key players in inhibition of apoptosis and autophagy [5-7].

Bcl-2, the prototypic prosurvival Bcl-2 family member which is associated with the translocation t(14;18) characteristic for follicular lymphoma was discovered in 1985 [8]. Since then more than 25 pro- and anti-apoptotic Bcl-2 proteins have been detected and characterized in regard to their clinical relevance in a repertory of different cancers [9]. Overexpression of pro-survival Bcl-2 family member proteins has been associated with poor chemotherapeutic response in bladder cancer [10,11]. In prostate cancer and glioblastoma, high expression of prosurvival Bcl-2 proteins has been shown to be correlated to apoptosis resistance and the propensity to induce an autophagy-dependent type of cell death [5,12].

The term autophagy refers to an evolutionarily conserved process in which intracellular proteins and organelles are sequestered in autophagosomes that represent specialized double-membrane containing vacuoles. Autophagosomes are subsequently targeted to lysosomes where their content is degraded by lysosomal enzymes for the purpose of recycling cellular components to sustain metabolism during nutrient deprivation and to prevent accumulation of damaged proteins and organelles [13,14]. Autophagy is a dynamic process, consisting of several sequential stages (initiation, nucleation, elongation, and maturation) controlled by a group of autophagy-related genes (ATG genes) that function in a hierarchical manner during the different stages of autophagosome biogenesis. ATG5, first discovered in yeast, is a core autophagy protein involved in the early stages of autophagosome formation [15]. In regard to cell death/survival decisions, the role of autophagy is highly contextual. In general autophagy acts as a pro-survival stress response, but it is hypothesized that it can also trigger cytodestructing effects. In line with this notion, there is evidence that overactivation of autophagy can act as an alternative cell death pathway [4,5,16-19].

Small-molecule inhibitors of prosurvival Bcl-2 proteins binding to their respective hydrophobic BH3 grooves, also termed BH3 mimetics, are capable of activating both apoptosis and autophagy [20,21]. AT-101, the (−) enantiomer of Gossypol, a natural product from cottonseed, has been identified as a small-molecule pan-Bcl-2 inhibitor, inactivating Bcl-2, Bcl-xL, Bcl-w and Mcl-1 [20,21]. (−)-gossypol exhibits cell death-promoting effects in various *in vivo* and *in vitro* cancer models, especially in those with an intact apoptotic machinery [22,23]. Prior studies have also shown that (−)-gossypol induces autophagy through release of the pro-autophagic molecule Beclin-1 from Bcl-2, thereby activating the autophagy pathway [12].

Here we investigated the response of chemosensitive and chemoresistant bladder cancer cells to treatment with the BH3 mimetic (−)-gossypol and the potential role of autophagy in this context. Our data show for the first time that chemoresistant cells are susceptible to treatment with (−)-gossypol, but exhibit an enhanced basal and drug-induced autophagy, which is associated with diminished apoptotic cell death. Our results suggest that enhanced autophagy may play an important role for the chemoresistant phenotype of bladder cancer.

## Methods

### Materials

The pan Bcl-2 inhibitor (−)-gossypol (>98% purity) was acquired from Tocris (Bristol, United Kingdom). Autophagy inhibitors Bafilomycin A1 (BafA1), and 3-Methyladenin (3-MA) were obtained from Sigma-Aldrich (Taufkirchen, Germany). The pan-caspase inhibitor z-Val-Ala-DL-Asp(OMe)-fluoromethylketone (z-VAD) was purchased from Bachem (Weil am Rhein, Germany). The Mcl-1 sparing Bcl-2 inhibitor ABT-737 was from Santa Cruz Biotechnology (Heidelberg, Germany) and the inductor of apoptotic cell death staurosporine (STS) was from Alexis Biochemicals (by ENZO Life Sciences, Lörrach, Germany). Chemotherapeutic gemcitabine was from Fresenius-Kabi (Bad Homburg, Germany) and chemotherapeutic cisplatin from Teva (Ulm, Germany). Lysotracker Red DND-99 was obtained from Invitrogen (Karlsruhe, Germany). All other chemicals were used in analytic grade purity from Sigma-Aldrich.

### Cell lines and culture

For this study, the parental chemosensitive human malignant bladder cancer cell lines 5637, which originates from a grade II bladder transitional cell carcinoma, and RT4, derived from a well differentiated grade I papillary bladder cancer, were obtained from ATCC/LGC Promochem GmbH (Wesel, Germany). Chemoresistant cell lines were established by continuous exposure of parental cells to increasing concentrations of the respective drug as described before [24-26]. The cisplatin (CDDP) and gemcitabine (GEMCI) resistant sublines were cultivated for more than 6 month under the continuous presence gemcitabine 20 ng/ml or cisplatin 1000 ng/ml. The cells were named following the published nomenclature, i.e. 5637^r^GEMCI^20^ means 5637 adapted to gemcitabine 20 ng/ml, 5637^r^CDDP^1000^ means 5637 adapted to cisplatin 1000 ng/ml. All cell lines were grown in IMDM supplemented with 10% fetal calf serum, 2% glutamine, and 1% penicillin/streptomycin (all: Gibco/Invitrogen, Karlsruhe, Germany) and cultivated in a humidified incubator at 37°C and 5% CO_2_.

### Ethics statement

Primary human material was not used in this study. All work presented has been performed in established, commercially available cell lines or derivates of these lines.

### MTT assay

Cells were seeded in 96-well-plates at 2,000 per well and cultivated for 24 h before onset of the treatment. After treatment, 20 μl of the 3-(4,5-dimethylthiazol-2-yl)- 2,5-diphenyltetrazolium bromide (MTT) stock solution (5 mg/mL) were added to 100 μL of medium in each well, followed by incubation at 37°C with a 5% CO_2_ atmosphere for 3 h. Following incubation, the medium containing the MTT reagent was removed, cells were solubilized by adding n-propyl alcohol/1 mol/L HCl 24:1), and lysates were gently mixed for 30 min. The absorbance at 560 nm were measured with a HTS fluorescent plate reader.

### SDS-PAGE and western blotting

Thirty micrograms (30 μg) of whole cell lysate [lysis buffer: 68.5 mM Tris/HCl pH 6.8, 2% sodium dodecyl sulfate (SDS), 10% glycerol, and protease inhibitors] was loaded onto 12 or 15% SDS–polyacrylamide gels. Proteins were separated at 120 V and then blotted to nitrocellulose membranes (Protean BA 83; 2 lm; Schleicher & Schuell, Dassel, Germany) in Towbin-buffer (25 mM Tris, 192 mM glycine and 20% methanol (v/v)) at 15 V for 35 min. The blots were blocked in blocking buffer (5% non-fat milk, 50 mM Tris–HCl pH 7.5, 150 mM NaCl and 0.05% Tween-20) at 20°C for 2 h. The resulting blots were probed with a mouse monoclonal anti–Bcl-2 antibody diluted 1:1,000 (Santa Cruz Biotechnology), a rabbit polyclonal anti– Bcl-xL antibody diluted 1:1,000 (Cell Signaling by New England Biolabs GmbH, Frankfurt, Germany), a rabbit polyclonal anti–Mcl-1 antibody diluted 1:1,000 (Cell Signaling), a rabbit polyclonal anti–Bcl-w diluted 1:1,000 (Cell Signaling) a rabbit polyclonal anti-ATG5 antibody (Cell Signaling) diluted 1:1,000, a rabbit polyclonal anti-Bax antibody diluted 1:500 (Upstate by Merck Millipore, Schwalbach, Germany), a rabbit polyclonal anti-Bak antibody diluted 1:1,000 (Santa Cruz Biotechnology), a mouse monoclonal anti-p62 diluted 1:1,000 (BD Bioscience, Heidelberg, Germany), a mouse monoclonal anti-LC3 antibody (Sigma-Aldrich) diluted 1:1,000, and a mouse monoclonal anti–glyceraldehyde-3-phosphate dehydrogenase (GAPDH) antibody (Calbiochem, Darmstadt, Germany) diluted 1:10,000.

### Flow cytometry

Cell death was detected by flow cytometry after Annexin V-Fluos/propidium iodide (PI) double staining (Roche Applied Science, Penzberg, Germany/Sigma-Aldrich). All cells that were positive for Annexin V and/or PI [i.e., cells from all quadrants except the bottom left one (Q3)] were considered dead. In all cases, a minimum of 10^4^ events per sample was acquired.

To quantitatively detect changes in activation of the autophagosomal/lysosomal pathway, acidic vacuoles were stained with 25 nmol/L Lysotracker Red DND-99 (Invitrogen) for 30 minutes and washed twice with PBS, and the net amount of acidic vesicles was determined by flow cytometric analyses. In all cases, a minimum of 10^4^ events per sample was acquired. Flow cytometric analyses were done on a FACS- Canto II (BD Biosciences) followed by analysis using FACSDiva software (BD Biosciences).

### Lentiviral transduction

Lentiviral vector stocks specific for ATG5 (SHVRS-NM_004849, Sigma Aldrich) were used for transduction of bladder cancer cells. The target sets included five sequences for different small hairpins. The pLKO.1-puro control transduction particles (SHC001V) did not contain a hairpin insert and were used as a negative control. For the transduction, cells were plated in 96 well plates and transduced the following day at a multiplicity of infection of 10. New medium was added to a final volume of 100 μL containing hexadimethrine bromide (Sigma-Aldrich) at a final concentration of 8 μg/mL. Cells were incubated for 24 hours before changing the medium. After overnight incubation, cells were washed, trypsinised and transferred to six-well plates, and further cultivated in medium containing 5 μg/μL puromycin (Calbiochem).

### Transient transfection and confocal microscopy

The cells were seeded on 13-mm coverslips, cultured for 24 hours. Next day cells were transfected with the expression plasmids mRFP-GFP-LC3 [27] using Metafectene (Biontex, Martinsried, Germany) reagent according to the manufacturer’s instructions. Twenty-four hours after transfection, cells were subjected to the respective treatment as indicated. Afterwards cells were fixed with paraformaldehyde (4% PFA, 4% sucrose), permeabilized with 0.1% Triton X100, stained with DAPI (AppliChem, Darmstadt, Germany), mounted on microscope slide and finally analyzed using a Nikon C1i confocal microscope. The fluorescence of GFP, RFP and DAPI was recorded with the suitable filter sets (GFP fluorescence: excitation 488 nm, emission 509 nm; RFP: excitation 554 nm, emission 568 nm, DAPI: excitation 358 nm, emission 461 nm). Digital images were obtained using EZ-C1 Nikon software. 100 cells from three different cultures were counted for each treatment (300 total).

### Statistics

Data are given as means ± SEM. For statistical comparison, one-way ANOVA followed by Tukey’s test was used using SPSS software (SPSS GmbH Software). P values <0.05 were considered to be statistically significant.

## Results

### (−)-Gossypol induces an apoptotic type of cell death in bladder cancer cells

**Figure 1:**
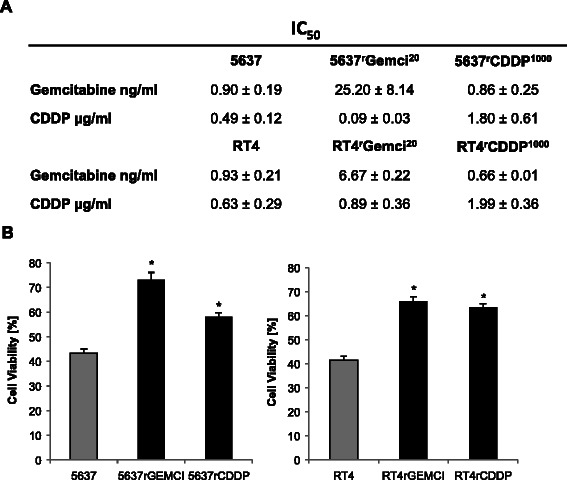
**Establishment of cellular resistance models.** Bladder cancer cell lines 5637 and RT4 were adapted to growth in the chemotherapeutic presence of 20 ng/ml gemcitabine (^r^GEMCI^20^) or 1000 ng/ml Cisplatin (^r^CDDP^1000^). 50% inhibiting concentration (IC_50_) Values are mean from three independant experiments ± SD and are shown in **A**. Chemo-sensitive (5637, RT4) and chemo-resistant cells (5637^r^GEMCI^20^, 5637^r^CDDP^1000^, RT4^r^GEMCI^20^, RT4^r^CDDP^1000^) were treated with 3 μM Staurosporin (STS), an inducer of apoptotic cell death, for 6 h and cell viability was measured by the MTT Assay.*, P < 0.05 compared with the parental cell line **(B)**.

Cisplatin and gemcitabine are chemotherapeutic agents which are also known for provoking alteration in the expression levels of Bcl-2 proteins [28,29]. In our study cells were adapted to growth in the presence of 20 ng/ml cisplatin and 1000 ng/ml gemcitabine for more than 6 months, and IC_50_ values for the effects of gemcitabine and cisplatin were obtained. Figure 1A provides a profile of the IC_50_ values for the effects of gemcitabine and cisplatin cross resistance on the viability of the investigated bladder cancer cell lines 5637, 5637^r^GEMCI^20^, 5637^r^CDDP^1000^, RT4, RT4^r^GEMCI^20^ and RT4^r^CDDP^1000^. In order to address the potential effects of acquired chemoresistance on the general sensitivity to apoptosis, we initially applied staurosporine, a well-established inducer of apoptotic cell death, and analyzed its effects on cell viability in chemosensible and chemoresistant 5637 and RT4 cells by MTT assays. STS caused a stronger decrease in cancer cell viability in parental 5637 and RT4 cells than in the cisplatin and gemcitabine chemoresistant 5637 and RT4 cell lines, respectively (Figure 1B).

**Figure 2:**
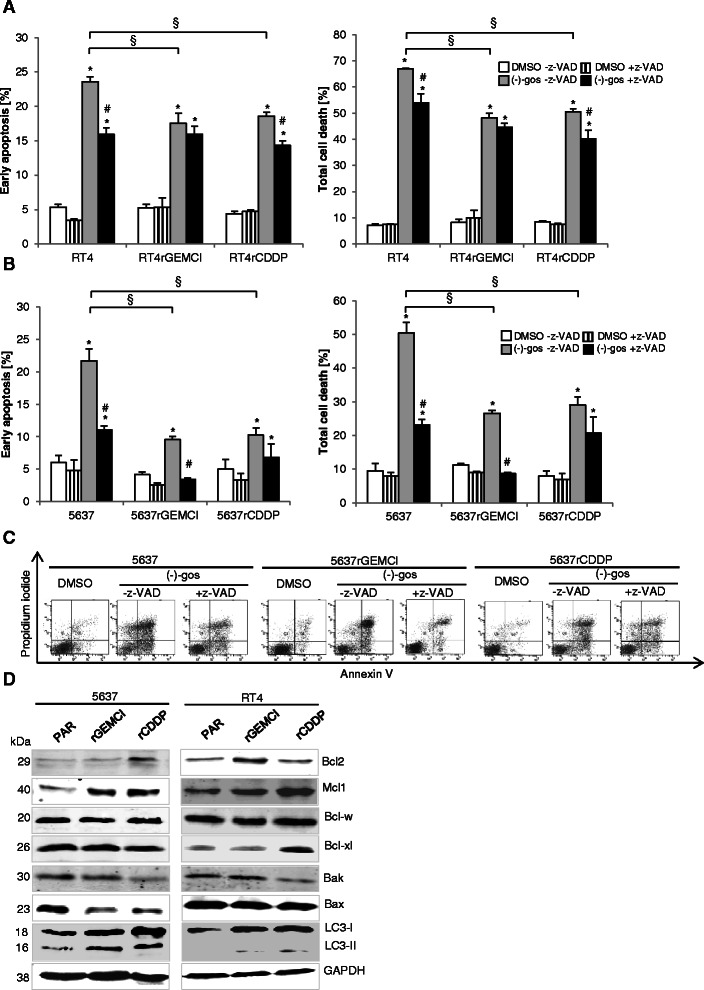
**(−)-Gossypol induces a caspase-dependent type of cell death in bladder cancer cells that is attenuated in gemcitabine- and cisplatin-resistant cells.** 5637, 5637^r^GEMCI^20^, 5637^r^CDDP^1000^, RT4, RT4^r^GEMCI^20^ and RT4^r^CDDP^1000^ cells were pre-treated with pan-caspase inhibitor z-VAD (100 μM) for 1 h followed by treatment with 10 μM of pan Bcl-2 inhibitor (−)-gossypol for 48 h. Total cell death and early apoptotis cell were quantified by flow cytometric analysis. *, P < 0,05 compared with the control. #, P > 0.05 compared to with or without z-VAD. §, P > 0.05 compared to the parental cell **(A and B)**. Representative data from the experiment B in 5637, 5637^r^GEMCI^20^ and 5637^r^CDDP^1000^ exhibited as FACS dot plot profiles **(C)**. Western blot analysis of the expression of pro-survival and pro-apoptotic Bcl-2 family members and LC-3 I and LC-3 II in 5637 parental (PAR), 5637^r^GEMCI^20^, 5637^r^CDDP^1000^, RT4 parental, RT4^r^GEMCI^20^ and RT4^r^CDDP^1000^cells. GAPDH served as a loading control **(D)**.

The natural BH3 mimetic (−)-gossypol has been described as a pan Bcl-2 inhibitor targeting Bcl-2, Bcl-xL, Mcl-1 and Bcl-w [5,21]. To evaluate the antitumor activity of (−)-gossypol in chemosensitive vs chemoresistant bladder cancer cells, we carried out FACS analysis of Annexin V binding and propidium iodide uptake to determine both early apoptosis and total cell death (Figure 2A and B). (−)-gossypol induced lower levels of early apoptosis and total cell death in cisplatin- and gemcitabine-chemoresistant RT4 and 5637 cells compared to parental bladder cancer cell lines 5637 and RT4. Resistance to cell death was more pronounced in the chemoresistant 5637 cell lines (Figure 2B) compared to RT4 cells (Figure 2A) with ~25% of dead cells in 5637^r^GEMCI^20^ and ~30% in 5637^r^CDDP^1000^ compared to ~50% in 5637 parental cells. RT4^r^GEMCI^20^ and RT4^r^CDDP^1000^ cells were also significantly protected from cell death compared to parental RT4 cells. Pre-treatment with z-VAD, a cell-permeant pan-caspase inhibitor that binds irreversibly to the catalytic site of caspase proteases, diminished cell death in 5637 and RT4 parental cells, and to a lesser degree in the chemoresistant RT4 cell lines. Analysis of protein expression levels of prosurvival and proapoptotic Bcl-2 family members revealed a pronounced increase of prosurvival Bcl-2 in 5637^r^CDDP^1000^ and RT4^r^GEMCI^20^. A higher level of Mcl-1 was detected in gemcitabine- and cisplatin-resistant cells both in 5637 and RT4 cells. The cell lines express similar protein levels of Bcl-w. Bcl-xL is increased in RT4^r^CDDP^1000^. Proapoptotic proteins Bak and Bax show a lower expression pattern in the chemoresistant lines 5637^r^GEMCI^20^, 5637^r^CDDP^1000^, RT4^r^GEMCI^20^ and RT4^r^CDDP^1000^. The chemoresistant lines 5637^r^GEMCI^20^, 5637^r^CDDP^1000^, RT4^r^GEMCI^20^ and RT4^r^CDDP^1000^ also revealed an increased amount of LC3-II, indicating an increase of basal autophagy.

Collectively, these results show that (−)-gossypol induces a caspase-dependent apoptotic type of cell death in parental bladder cancer cells which is partially inhibited in chemoresistant cells expressing higher levels of anti-apoptotic and lower levels of pro-apoptotic Bcl-2 family members and also exhibiting markers of enhanced basal autophagy.

### Suppression of autophagy potentiates (−)gossypol-induced cell death only in chemoresistant bladder cancer cells

**Figure 3:**
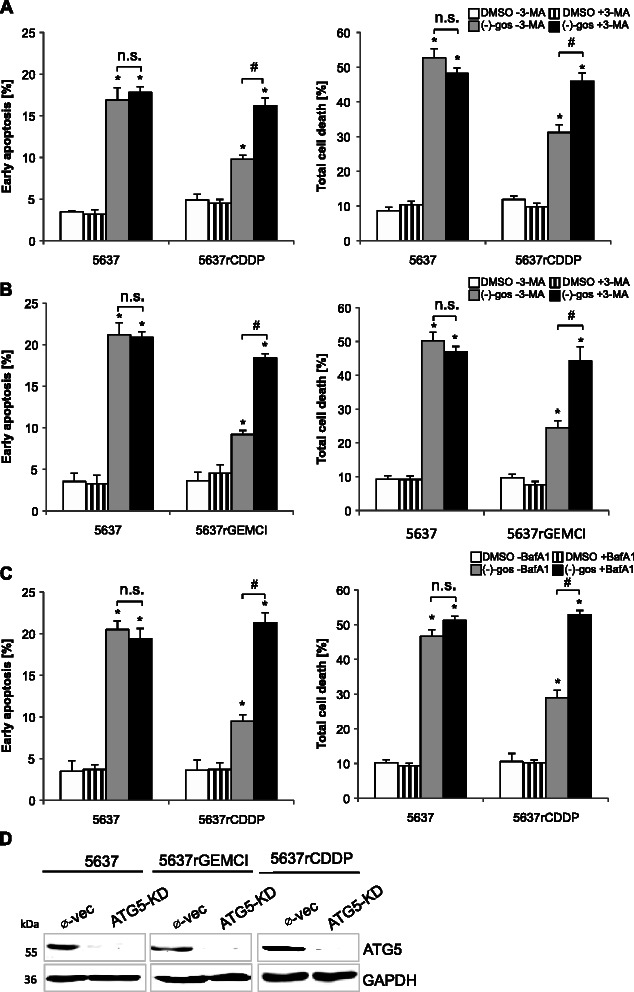
**Pharmacological inhibition of autophagy potentiates (−)-gossypol-induced cell death in chemoresistant bladder cancer lines.** Bladder cancer cells 5637, 5637^r^CDDP^1000^ and 5637^r^GEMCI^20^ were treated with 15 μM of pan Bcl-2 inhibitor (−)-gossypol with or without the autophagy inhibitor 3-MA (2 mM) for 48 h and total cell death and apoptotic cell death were quantified by flow cytometry of Annexin V/PI double staining **(A and B)**. *, P < 0.05, compared with the control. #, P < 0.05, compared with cultures not cotreated with 3-MA. n.s., not significant. The inhibitor of autophagic flux Bafilomycin A1 potentiates cell death induced by (−)-gossypol in 5637^r^CDDP^1000^ cells **(C)**. Bladder cancer cells 5637 and 5637^r^CDDP^1000^ were treated with 15 μM (−)-gossypol with or without Bafilomycin A1 (10 nM) for 48 h and total cell death and apoptotic cell death was quantified by flow cytometry of Annexin V/PI double staining. *, P < 0.05, compared with the control. #, P < 0.05, compared with cultures not co-treated with Bafilomycin A1. n.s., not significant. Stable knockdown of ATG5 in 5637, 5637^r^GEMCI^20^ and 5637^r^CDDP^1000^ cells as shown by Western blot analysis with an ATG5 antibody **(D)**. Whole-cell lysates of 5637, 5637^r^GEMCI^20^ and 5637^r^CDDP^1000^ control cells transfected with empty vector (Ø-vec) and ATG5-KD cells were probed and analyzed with an antibody against ATG5. GAPDH served as a loading control.

In light of the fact that (−)-gossypol can induce cytoprotective autophagy in apoptosis-proficient cancer cells [30], but an autophagy-dependent type of cell death in apoptosis-deficient cancers such as malignant glioma and prostate cancer [5,12], we wanted to investigate the potential cell death-modulatory function of autophagy in our bladder cancer models. When blocking autophagy with the conventional inhibitor 3-MA, (−)-gossypol-induced cell death was significantly increased in the chemoresistant 5637^r^CDDP^1000^ and 5637^r^GEMCI^20^ cells, whereas the amount of cell death remained largely unaltered in parental 5637 cells (Figure 3A and B). Similar results were obtained with another pharmacological inhibitor of autophagic flux Bafilomycin A1, a specific V-ATPase inhibitor (Figure 3C). These results demonstrate that autophagy may be causally related to the acquired chemotherapy resistance of the investigated cell lines.

**Figure 4:**
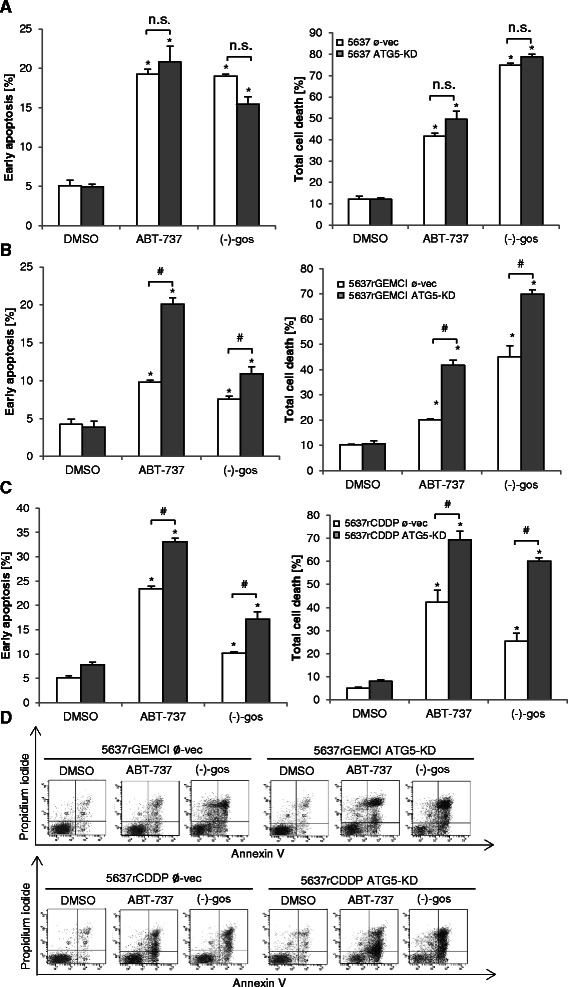
**Knockdown of ATG5 sensitizes chemoresistant 5637 cells to (−)-gossypol.** Quantification of total cell death and early apoptosis by flow cytometry (Annexin V/PI). Empty vector–transfected 5637, 5637^r^GEMCI^20^, 5637^r^CDDP^1000^ cells (Ø-vec) and 5637, 5637^r^GEMCI^20^, 5637^r^CDDP^1000^ ATG5-KD cells were treated with 10 μM of Mcl-1 sparing Bcl-2 inhibitor ABT-737 and 15 μM of pan Bcl-2 inhibitor (−)-gossypol for 48 h. *, P < 0.05 compared with the control. #, P > 0.05 compared with Ø-vec with the same respective treatment. n.s., not significant **(A-C)**. Representative data from the experiment B and C in 5637^r^GEMCI^20^ Ø-vec, 5637^r^GEMCI^20^ ATG5-KD and 5637^r^CDDP^1000^ Ø-vec, 5637^r^CDDP^1000^ ATG5-KD shown in **D** exhibited as FACS dot plot profiles.

ATG5 is involved in early stages of autophagosome formation [31], thus its knockdown causes interference with induction of macroautophagy. For further determination of the role of autophagy in cell death induced by BH3 mimetics, a stable ATG5 knockdown (ATG5-KD) was established in 5637, 5637^r^GEMCI^20^ and 5637^r^CDDP^1000^ cells (Figure 3D), after which cultures of these cells were treated with (−)-gossypol and ABT-737, which is capable of inhibiting Bcl-2, Bcl-xL, and Bcl-w, but not Mcl-1 [21]. After 48 h of (−)-gossypol and ABT-737 treatment the Annexin V positive/PI negative fraction representing the early apoptotic cells and overall cell death (double positive cells) were not significantly changed in 5637 ATG5-KD cells compared to the parental ones/cells, ATG5-proficient control (Figure 4A). In contrast, early apoptosis and total cell death were significantly enhanced in 5637^r^GEMCI^20^ ATG5-KD and 5637^r^CDDP^1000^ ATG5-KD chemoresistant cells after both treatments, indicating a cytoprotective function of autophagy (Figure 4B and C).

### Enhanced basal and (−)-gossypol-induced autophagy in chemoresistant bladder cancer cells

**Figure 5:**
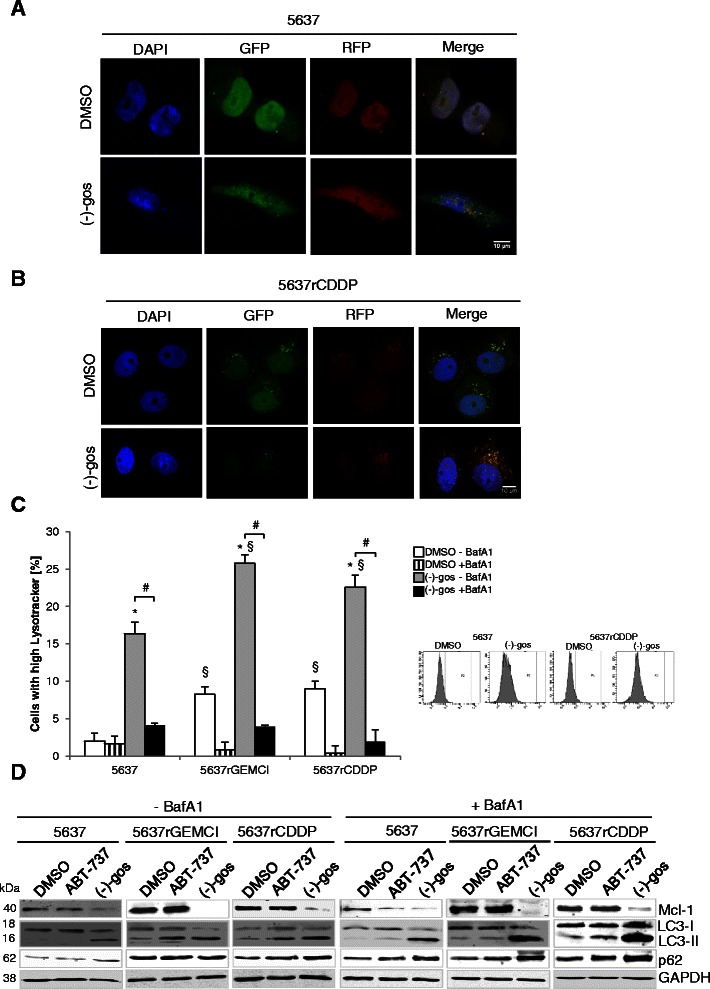
**Enhanced basal and drug-induced autophagy in chemoresistant 5637 cells.** Detection of the autophagic flux with the mRFP-GFP-LC3 tandem fluorescence construct by confocal microscopy (A and B). 5637 **(A)** and 5637^r^CDDP^1000^**(B)** cells were transiently transfected with mRFP-GFP-LC3 and treated with 15 μM of pan Bcl-2 inhibitor (−)-gossypol for 24 h. After fixation, nuclei were labeled with DAPI and digital images of representative cells were acquired. Bar, 10 μm. Quantification of lysosomal activity **(C)** Bladder cancer cells 5637, 5637^r^GEMCI^20^ and 5637^r^CDDP^1000^ were treated with 10 μM (−)-gossypol with or without Bafilomycin A1 (10 nM ) for 48 h and the net amounts of acidic vesicles in the cultures were measured by staining with Lysotracker Red DND-99 (25 nmol/L) and flow cytometry. *, P < 0.05, compared with the control. #, P < 0.05, compared with cultures not co-treated with inhibitor of autophagic flux Bafilomycin A1. §, P < 0.05 compared with the parenteral cell line. Western blot analysis of the expression of Mcl-1,LC-3 I, LC-3 II and p62 in 5637, 5637^r^GEMCI^20^ and 5637^r^CDDP^1000^ cells **(D)** Cells were treated with 10 μM of Mcl-1 sparing Bcl-2 inhibitor ABT-737 and 15 μM (−)-gossypol with or without Bafilomycin A1 (10 nM ) for 48 h. GAPDH served as a loading control.

To evaluate the role of autophagy in (−)-gossypol treated cells, we used an LC3 tandem fluorescence construct allowing to discriminate between autophagosomal and autolysosomal LC3 (1). 5637 and 5637^r^CDDP^1000^ cells were treated with 15 μM (−)-gossypol for 24 hours to monitor the autophagic flux at the single-cell level. Untreated 5637 cells displayed fewer bright, diffuse GFP and mRFP fluorescence signals than untreated 5637^r^CDDP^1000^. After treatment with (−)-gossypol 5637^r^CDDP^1000^ displayed enhanced formation of autophagosomes (colocalized fluorescence of GFP and mRFP) and a parallel increase of autolysosomes (only mRFP fluorescence). In 5637 cells the number of mRFP-positive autolysosomes under treatment with (−)-gossypol was much lower (Figure 5A and B).

We also stained acidic vacuoles with Lysotracker Red and subsequently measured the extent of acidic vesicles by FACS analysis (Figure 5C). Therefore the cells were treated with (−)-gossypol for 48 h. Additionally 10 nM Bafilomycin A1 was added 4 h before the end of the treatment to inhibit autophagic flux. Induction of autophagy is associated with a net increase in the percentage of cells displaying high Lysotracker fluorescence, as established for detection of (−)-gossypol-induced autophagy. The percentage of cells highly labeled with Lysotracker was significantly increased in both chemoresistant cell lines vs. the control, indicating enhanced basal autophagy. These observations were confirmed by Western blotting, indicating increased LC3 conversion under basal conditions (Figure 5D). In addition, both chemoresistant cell lines displayed an enhanced LC3 conversion after treatment with (−)-gossypol (Figure 5D) as well as elevated Lysotracker staining (Figure 5C) which was potently blocked by Bafilomycin A1. Figure 5D also shows that (−)-gossypol significantly lowers the levels of Mcl-1, possibly through targeting Mcl-1 for degradation [5].

## Discussion

Bladder cancer (urothelial cancer) is currently the fifth most commonly diagnosed type of cancer among men in the United States [1]. Cancer of the bladder is highly aggressive and treatment of metastatic disease has shown little or no efficiency. When the disease becomes metastatic, the median survival is 14 months, even with standard chemotherapy [32]. The first-line standards in chemotherapy include the chemotherapeutic agents gemcitabine and cisplatin [33]. Both agents exert anti-cancer effects via multiple mechanisms [34-36]. The antitumor activity gemcitabine and cisplatin varies even from patient to patient and the development of drug resistance are believed to be related to variations in intracellular drug metabolite levels and activities of drug transporters, drug metabolizing enzymes and target enzymes [37]. These factors and the cancer-associated genomic heterogeneity [38] that is inherent in tumor-derived cell lines seem to underlie much of the variability in the responses to chemotherapeutic agents as evident by the IC_50_ values. This phenomenon of marked differences in IC_50_ values of different cell lines of one tumor entity has already been observed in previous studies [39,40].

Another key mechanism of therapy escape is represented by the inability of these cells to undergo apoptosis, which underscores the importance of disturbed apoptotic signaling in cancer progression.

Proteins of the Bcl-2 family are pivotal regulators of apoptosis, but also represent important inhibitors or inducers of autophagy [6,7,41]. Bcl-2 family proteins play a central role in the intrinsic (mitochondrial) pathway of apoptosis, which activates increased mitochondrial membrane permeability and the release of pro-apoptotic molecules into the cytoplasm [42]. The Bcl-2 family consists of both anti-apoptotic and pro-apoptotic proteins. The pro-apoptotic molecules and pore-forming multidomain proteins Bax and Bak, as well as the BH3-only proteins Bad, Bim, Puma, Noxa and Bid couple diverse stress signals to the intrinsic apoptosis pathway. Anti-apoptotic members such as Bcl-2, Bcl-xL, Bcl-w and Mcl-1 are highly overexpressed in many malignancies including bladder cancer are known to adversely affect chemosensitivity and radiosensitivity [43,44]. Upon induction of the intrinsic pathway by BH3-only proteins, Bax and Bak are inserted into the outer mitochondrial membrane promoting outer mitochondrial outer membrane permeabilisation (MOMP) and cytochrome c release [42]. In contrast, the anti-apoptotic Bcl-2 family members block apoptosis by preventing Bax and Bak activation and MOMP [42]. In terms of chemoresistance, Wong et al. stated that gemcitabine resistance is associated with high Bcl-2 protein expression in different tumors. Gene expression profiling in gemcitabine resistant and gemcitabine sensible cell lines suggest that anti-apoptotic genes such as *Akt* and *PI3KR2* may play important role in gemcitabine resistance, while pro-apoptotic Bcl-2 related genes (*Bad, Caspase-6 and Calpain-1*) may regulate synergistic interaction in combination therapy [45]. Here studies have shown that qPCR techniques are not able to differentiate among the posttranslational modifications of individual members of the Bcl-2 subfamily at the protein level. In this regard, it has been reported that significant stabilization or proteolysis of Bcl-2 and Mcl-1 protein may be facilitated by phosphorylation [46]. Furthermore, the turnover of antiapoptotic Bcl-2 subfamily members has been reported to be dependent on several other proteins, including the levels of proapoptotic Bcl-2 subfamily members, the E3 ubiquitin ligase, MULE/LASU1, or individual caspases, to name a few [47].

Autophagy is an evolutionarily conserved, pervasive and multi-step “self-eating” process, by which cytosolic material is sequestered in a double-layered membrane, delivered to the lysosome for degradation and digested to provide energy and building the basis for cell-survival [13]. Autophagy can trigger both cytoprotective and death-promoting effects and thus its role in modulation of cell death is highly contextual. Autophagy primarily serves to alleviate stress, but there is evidence that over activation of autophagy can also act as an alternative cell death pathway [4,48]. As indicated above, the anti-apoptotic members of the Bcl-2 family also function as anti-autophagic regulators via their inhibitory interaction with the core autophagy factor and non-apoptotic BH3-only protein Beclin 1 which is involved in autophagosome formation. It has been reported that Beclin 1 directly interacts with Bcl-2, Bcl-xL, Bcl-w and to a smaller extent with Mcl-1 [49].

To analyze the potential therapeutic relevance of Bcl-2-targeted therapy and mechanisms promoting cell death resistance in chemoresistant bladder cancer, we established cisplatin- and gemcitabine-resistant cell lines. These two chemotherapeutic drugs are also known for possible modulatory effects on Bcl-2 protein levels. In endometrial cancer cells, cisplatin increases Bcl-2 expression via activation of protein kinase C and Akt2 [28]. Shi et al. observed that pancreatic cancer cells with acquired drug resistance to gemcitabine exhibit an upregulation of Bcl-xl and Mcl-1 [29]. In our study, cells were adapted to growth in the presence of cisplatin and gemcitabine for more than 6 months, giving rise to lines highly resistant to these drugs. Chemoresistant 5637 and RT4 cell lines exhibited an enhanced resistance to the apoptosis inductor staurosporine, supporting the notion that the intrinsic propensity of chemoresistant cells to undergo apoptosis is partially compromised in comparison to the chemosensitive cells.

For Bcl-2 targeting, we used two BH3 mimetics with different binding profiles to Bcl-2 family members. The BH3 mimetic ABT-737 can inactivate Bcl-2, Bcl-xL, and Bcl-w, but not Mcl-1 [20,21]. The natural pan-Bcl-2 inhibitor (−)-gossypol is capable of inhibiting the four major antiapoptotic Bcl-2 family members Bcl-2, Bcl-xL, Bcl-w and Mcl-1 [20,21]. Interestingly (−)-gossypol favorably induced an autophagic cell death in apoptosis-resistant cells with high levels of Bcl-2 and Bcl-xL whereas cell lines with lower expression were prone to (−)-gossypol-induced apoptosis [12].

As previously described for UM-UC bladder cancer cells [50] (−)- gossypol induced an apoptotic cell death in parental RT4 and 5637 bladder cancer cells as demonstrated by the fact that the pan-caspase inhibitors z-VAD could significantly block cell death. On a closer look at the RT4 and 5637 cell triplets, chemoresistant 5637^r^CDDP^1000^, 5637^r^GEMCI^20^ and RT4^r^GEMCI^20^ and RT4^r^CDDP^1000^ cells show a pronouncedly higher anti-apoptotic Mcl-1 expression levels than the chemosensitive cells. Similarly, Michels et al. have shown for CDDP-resistant lung cancer cells that only a minority of cell lines overexpressed Bcl-2 or Bcl-xL, but a manifested increase in Mcl-1 protein expression level was seen [51].

In general there are many lines of evidence demonstrating that the dysregulation of Bcl-2 family members, −especially the overexpression of anti-apoptotic Bcl-2 proteins in various malignancies (including those of the genitourinary tract)-, is crucial for not only the failure of standard therapy with the promotion of chemoresistance, but also correlates with progression of cancer, disease recurrence and disease specific mortality [11,43,44,52-57]. The potential mechanisms driving aberrant expression of Bcl-2 proteins in tumor cells are diverse and may include gene amplification, epigenetic changes, posttranslational modifications as well as overactivation of upstream transcription factors (e.g. NFκB. STAT3).

In terms of pro-apoptotic proteins Bak and Bax, chemoresistant 5637^r^CDDP^1000^, 5637^r^GEMCI^20^ and RT4^r^GEMCI^20^ and RT4^r^CDDP^1000^ cells show a lower expression pattern than in the chemosensitive cells. Wang et al. have stated that Bak-deficient T leukemic cells were resistant to apoptosis induced by various anticancer drugs [58]. In line with this evidence Balakrishnan et al. demonstrate that Bak and Bax are required for apogossypolone to induce apoptosis [59].

Similar to their response to STS, chemoresistant 5637 and RT4 cells displayed a lower sensitivity to (−)-gossypol treatment as determined by quantification of early apoptosis and overall cell death. In comparison to chemonaive cells, the limiting effects of z-VAD on cell death were less pronounced in 5637^r^CDDP^1000^ and RT4^r^GEMCI^20^ cells, indicating the existence of an intact apoptosis machinery in these cells. Of note, (−)-gossypol was shown to induce a predominantly apoptotic cell death in cells with low Bcl-2 [12]. In line with this finding, the expression levels of Bcl-2 were significantly enhanced in 5637^r^CDDP^1000^ vs 5637^r^GEMCI^20^ and RT4^r^GEMCI^20^ vs. RT4^r^CDDP^1000^ cells.

In RT4^r^GEMCI^20^ and 5637^r^CDDP^1000^ cells, z-VAD did not elicit any effects on (−)-gossypol-induced cell death, suggesting induction of an alternative, caspase-independent type of cell death. Prior studies have shown that (−)-gossypol induces autophagy through release of the pro-autophagic molecule Beclin-1 from Bcl-2, thereby activating the autophagy pathway [12]. In line with these observations, we have previously demonstrated that (−)-gossypol induces an autophagy-dependent cell death in malignant glioma cells highly overexpressing Bcl-2, Bcl-xL and Mcl-1 [5].

The chemoresistant lines 5637^r^GEMCI^20^, 5637^r^CDDP^1000^, RT4^r^GEMCI^20^ and RT4^r^CDDP^1000^ revealed an increased amount of LC3-II, indicating an enhanced basal autophagy. In line with this, Bao et al. also proposed that the induction of autophagy contributes to cisplatin resistance in human ovarian cancer [60].

Despite this overwhelming evidence for the cytoprotective function of autophagy, the role of autophagy in drug resistance remains controversial. Accordingly, the data from another study suggest that long-term exposure to cisplatin caused an acquired drug resistance through a mechanism involving autophagy reduction in human lung cancer cells [61].

In order to define the potential cell death-modulating role of autophagy in bladder cancer cells, we applied the pharmacological autophagy inhibitors 3-MA and Bafilomycin A1. In contrast to glioma, inhibition of autophagy had no discernible effect on cell death in chemonaive 5637 cells. However, inhibition of autophagy with both compounds led to an *increase* in cytotoxicity and overall cell death in both chemoresistant 5637 lines, suggesting that autophagy may contribute to the enhanced resistance in these cells. This death-enhancing effect was also observed when we blocked autophagy by ATG5 siRNA in both resistant lines after treatment (−)-gossypol and ABT-737. These results suggest an “autophagy addiction” of chemoresistant bladder cancer cells, and a protective role of autophagy as previously demonstrated in other tumor cells subjected to (−)-gossypol treatment [30,62].

In line with our hypothesis of an “autophagy addiction” in the chemoresistant cells, untreated 5637^r^CDDP^1000^ cells exhibited characteristic signs of enhanced autophagy, with a higher basal LC3 conversion/translocation and autophagosomal/lysosomal activity than the 5637 parental cells. This effect on basal autophagy could be significantly blocked by Bafilomycin A1. After treatment with (−)-gossypol, cell death in 5637 parental cells was associated with nuclear condensation and cell shrinkage, but noticeable translocation of GFP-LC3 to autophagosomes occured only in a minor fraction of cells. In contrast, our study shows for the first time that (−)-gossypol further enhanced autophagosomal/lysosomal activity in chemoresistant cancer cells displaying elevated translocation of GFP-LC3 to autophagosomes, and ensuing autophagic flux of LC3 to autolysosomes.

The prosurvival function of autophagy has been linked to its ability to suppress various forms of cell death, including apoptosis. As mentioned before, (−)-gossypol can induce cytoprotective autophagy but the critical determinants for cells to be driven towards cytoprotective vs cytotoxic autophagy in response to (−)-gossypol treatment remain unclear. In light of the important role of mitochondria in controlling the fate of cell, it appears reasonable to speculate that upon (−)-gossypol treatment, self-defensive autophagy occurs to ensure the turnover of damaged mitochondria [30], but enhanced mitochondrial damage can reach levels above which excessive autophagy occurs and is followed by cell death. Therefore, the degree of mitochondrial damage and the capability of cells to deal with damaged mitochondria appear to contribute to determine which type of autophagy will occur upon gossypol treatment [63].

## Conclusion

We demonstrate for the first time that (−)-gossypol simultaneously induces apoptosis and a cytoprotective form of autophagy in bladder cancer. However, this (basal and drug-induced) cytoprotective type of autophagy is only evident after acquisition of chemotherapy resistance, arguing for a potential resistance mechanism acquired during the selection process of tumor cells. Therefore, enhanced autophagy may represent an “addiction” to autophagy, which may be therapeutically exploited. Targeting Bcl-2 in combination with inhibitors of autophagy in chemoresistant cancer shows a possible route for new strategies to overcome resistance to current cancer therapy.

## Abbreviations

BafA1: Bafilomycin A1
3-MA: 3-Methyladenin
z-VAD: Z-Val-Ala-DL-Asp(OMe)- fluoromethylketone
STS: Staurosporine
PI: Propidium iodide
PAR: Parental
CDDP: Cisplatin
GEMCI: Gemcitabine
MTT: 3-(4,5-dimethylthiazol-2-yl)- 2,5-diphenyltetrazolium bromide
MOMP: Mitochondrial outer membrane permeabilisation
